# Study protocol: effects of treatment expectation toward repetitive transcranial magnetic stimulation (rTMS) in major depressive disorder—a randomized controlled clinical trial

**DOI:** 10.1186/s13063-023-07579-4

**Published:** 2023-08-24

**Authors:** Katharina M. Steiner, Dagmar Timmann, Ulrike Bingel, Angelika Kunkel, Tamas Spisak, Manfred Schedlowski, Sven Benson, Harald Engler, Norbert Scherbaum, Katja Koelkebeck

**Affiliations:** 1 Department of Psychiatry and Psychotherapy, Medical Faculty, LVR-University-Hospital Essen, University of Duisburg-Essen, Virchowstr, 174, 45147 Essen, Germany; 2 Department of Neurology, University Hospital Essen, University of Duisburg-Essen, Essen, Germany; 3 Center for Translational Neuro- & Behavioral Sciences (C-TNBS), University Duisburg Essen, Essen, Germany; 4 Institute of Medical Psychology and Behavioral Immunobiology, University Hospital Essen, University of Duisburg-Essen, Essen, Germany; 5 Institute for Medical Education, Essen University Hospital, University Duisburg-Essen, Essen, Germany

**Keywords:** Major depressive disorder (MDD), Transcranial magnetic stimulation (TMS), Treatment expectation, Cerebellum, Resting-state fMRI

## Abstract

**Background:**

Patients’ expectations toward any given treatment are highly important for the effectiveness of such treatment, as has been demonstrated for several disorders. In particular, in major depressive disorder (MDD), one of the most frequent and most serious mental disorders with severe consequences for the affected, the augmentation of available treatment options could mean a ground-breaking success. Repetitive transcranial magnetic stimulation (rTMS), a new, non-invasive, and well-tolerated intervention with proven effects in the treatment of MDD, appears particularly suitable in this context as it is assumed to exert its effect via structures implicated in networks relevant for both expectation and depression.

**Methods:**

All patients will receive rTMS according to its approval. Half of the patients will be randomized to a psychological intervention, which is a comprehensive medical consultation aiming to improve positive treatment expectations; the control group will receive a conventional informed consent discussion (in the sense of a treatment-as-usual condition). As outcome parameters, instruments for both self-assessment and external assessment of depression symptoms will be applied. Furthermore, psycho-immunological parameters such as inflammation markers and the cortisol awakening response in saliva will be investigated. Resting-state functional magnetic resonance imaging (rs fMRI) will be performed to analyze functional connectivity, including the cerebellum, and to identify neuronal predictors of expectation effects. In addition, possible cerebellar involvement will be assessed based on a cerebellar-dependent motor learning paradigm (i.e., eyeblink conditioning).

**Discussion:**

In this study, the effects of treatment expectations towards rTMS are investigated in patients with MDD. The aim of this study is to identify the mechanisms underlying the expectation effects and, beyond that, to expand the potential of non-invasive and well-tolerated treatments of MDD.

**Trial registration:**

German Registry of Clinical Studies (DRKS DRKS00028017. Registered on 2022/03/07. URL: https://www.drks.de/drks_web/.

## Administrative information

**Table:** 

Title {1}	Study protocol: effects of treatment expectation toward repetitive transcranial magnetic stimulation (rTMS) in Major depressive disorder—a randomized controlled clinical trial
Trial registration {2a} and {2b}	German Registry of Clinical Studies (DRKS) Registration number: DRKS00028017, registration date: 2022/03/07 URL: https://www.drks.de/drks_web/
Protocol version {3}	Issue Date: 11 July 2022 Trial protocol version: 1
Funding {4}	This study is funded by the German Research Association (DFG), the Federal Ministry of Education and Research Germany (BMBF), and the Medical Faculty of the University Duisburg-Essen [Clinician Scientist Academy of the University Hospital Essen (UMEA)]. This study is associated to and supported by the transregional DFG Collaborative Research Centre (CRC/Transregio 289) “Treatment Expectation.”
Author details {5a}	Katharina M. Steiner (corresponding author, first author): LVR-University-Hospital Essen, Department of Psychiatry and Psychotherapy, Medical Faculty, University of Duisburg-Essen, Essen, Germany, Department of Neurology, University Hospital Essen, University of Duisburg-Essen, Essen, Germany, Center for Translational Neuro- & Behavioral Sciences (C-TNBS), University Duisburg Essen, Germany Dagmar Timmann: Department of Neurology, University Hospital Essen, University of Duisburg-Essen, Essen, Germany, Center for Translational Neuro- & Behavioral Sciences (C-TNBS), University Duisburg Essen, Germany Ulrike Bingel: Department of Neurology, University Hospital Essen, University of Duisburg-Essen, Essen, Germany, Center for Translational Neuro- & Behavioral Sciences (C-TNBS), University Duisburg Essen, Germany Angelika Kunkel: Department of Neurology, University Hospital Essen, University of Duisburg-Essen, Essen, Germany, Center for Translational Neuro- & Behavioral Sciences (C-TNBS), University Duisburg Essen, Germany Tamas Spisak: Department of Neurology, University Hospital Essen, University of Duisburg-Essen, Essen, Germany, Center for Translational Neuro- & Behavioral Sciences (C-TNBS), University Duisburg Essen, Germany Manfred Schedlowski: Center for Translational Neuro- & Behavioral Sciences (C-TNBS), University Duisburg Essen, Germany, Institute of Medical Psychology and Behavioral Immunobiology, University Hospital Essen, University of Duisburg-Essen, Germany Sven Benson: Center for Translational Neuro- & Behavioral Sciences (C-TNBS), University Duisburg Essen, Germany, Institute of Medical Psychology and Behavioral Immunobiology, University Hospital Essen, University of Duisburg-Essen, Germany, Institute for Medical Education, Essen University Hospital, University Duisburg-Essen, Ger-many Harald Engler: Center for Translational Neuro- & Behavioral Sciences (C-TNBS), University Duisburg Essen, Germany, Institute of Medical Psychology and Behavioral Immunobiology, University Hospital Essen, University of Duisburg-Essen, Germany Norbert Scherbaum: LVR-University-Hospital Essen, Department of Psychiatry and Psychotherapy, Medical Faculty, University of Duisburg-Essen, Essen, Germany, Center for Translational Neuro- & Behavioral Sciences (C-TNBS), University Duisburg Essen, Germany Katja Koelkebeck: LVR-University-Hospital Essen, Department of Psychiatry and Psychotherapy, Medical Faculty, University of Duisburg-Essen, Essen, Germany, Center for Translational Neuro- & Behavioral Sciences (C-TNBS), University Duisburg Essen, Germany
Name and contact information for the trial sponsor {5b}	This is a monocentric, investigator-initiated study Trial sponsor: LVR-University-Hospital Essen, Department of Psychiatry and Psychotherapy, Medical Faculty, University of Duisburg-Essen, Essen, Germany, Center for Translational Neuro- & Behavioral Sciences (C-TNBS), University Duisburg Essen, Germany Address: LVR-University-Hospital Essen, Virchowstrasse 174, 45147 Essen Email: katharinamarie.steiner@uk-essen.de
Role of sponsor {5c}	This is a monocentric, investigator-initiated study KK and DT are chief investigators. They conceptualized the study and developed the protocol together with KMS, who is an investigator. NS reviewed the manuscript and enabled the implementation of TMS in the LVR-University-Hospital Essen. UB, MS, SB, and HE are cooperation partners; UB: MRI; MS and SB: cognitive tasks; MS and HE: biological parameters. All authors read and approved the final manuscript

## Introduction

### Background and rationale {6a}

Major depressive disorder (MDD) is one of the most frequent—and most serious—disorders with a lifetime prevalence of up to 20%. Besides cardiovascular diseases, it is the disease which causes the greatest individual suffering when regarding disability-adjusted life years (DALYs) and generates the highest public health costs in Western societies [1]. About one-third of patients meet the criteria for difficult-to-treat depression, also referred to as treatment-resistant depression [2], although the criteria applied here are sometimes inconsistent. In most cases, this refers to the group of patients who have not experienced a significant reduction of symptoms even after two attempts at drug therapy with sufficient dosage and treatment time. In view of this large number and the high level of suffering in depressive patients, there is an urgent need to improve the existing treatment options.

A new approach could be to influence a patient’s treatment expectations. This, of course, requires a deeper understanding of the underlying mechanisms. Expectations are essential for the success of a treatment, which was shown in the treatments of pain, anxiety, and Parkinson’s disease [3] in a study that found an overt treatment to be much more effective than a covert treatment which, due to its inherent concealment, renders smaller effects of expectation.

Expectations seem to play a major role in the treatment of MDD as there are pronounced placebo effects in studies regarding antidepressant medication [4]. Rutherford and colleagues [5] investigated overt vs. covert administration of citalopram and found that citalopram was only superior to placebo in cases of an overt treatment. Attempting to explain these effects, several studies argue that placebo and pharmacological effects cannot clearly be separated and that antidepressants exert their influence via interactions with the treatment context [6].

In MDD, in particular, a psychological intervention that was designed to enhance expectations was shown to improve the effect of antidepressant medication [7]. The intervention involved participants attending four weekly-held cognitive-psychoeducational group therapy sessions.

Today, the topic of treatment expectation may be of particularly great relevance, as patient-physician communication has taken a back seat in the clinical setting in the face of numerous bureaucratic and economic challenges. New insights into the impact of treatment expectations could drive significant change, for example, regarding the design of the patient education discussion.

Transcranial magnetic stimulation (TMS) therapy is approved for the treatment of depressed patients who did not sufficiently benefit from standard antidepressant pharmacotherapy; its effectiveness has been shown in several meta-analyses [8]. The stimulation site of TMS treatment is the left dorsolateral prefrontal cortex. It is usually applied for a duration of 4 to 8 weeks, 5 days a week for 30 min [8, 9]. To date, it remains unclear how exactly TMS exerts its effects. Resting-state (rs) functional imaging studies found characteristic alterations in MDD which can be influenced by TMS [10]. In MDD, a hyperconnectivity within the frontoparietal network (involving the dorsolateral prefrontal cortex and the posterior parietal cortex) and a hyperconnectivity within the default mode network (DMN, involving the prefrontal and cingulate cortex) have been reported [10]. After repetitive (r)TMS treatment, Philip and colleagues [11] found altered rs functional connectivity between the prefrontal cortex and the subgenual anterior cingulate cortex. Higher connectivity within the DMN seems to predict the beneficial effects of both pharmacotherapy and TMS.

Interestingly, these same networks are implicated in expectation effects: Burke and colleagues [12] conducted a meta-analysis on neuroimaging studies of placebo effects and compared clusters of activation (and deactivation) with TMS and deep brain stimulation (DBS) targets for depression treatment and found an extensive overlap. This raises both the question and the hypothesis: can we assume a bilateral interaction between placebo mechanisms and TMS?

Another interpretation of this observation could be that there seems to be an overlap of the underlying cognitive-affective and reward-based substrates in MDD and placebo effects [12].

The cerebellum has only recently been added as a part of the neural network underlying placebo effects [13]. Furthermore, newer studies suggest that the cerebellum is not only involved in the processing of sensory, but also reward predictions and prediction errors [14–16]. Several studies reported structural and functional alterations in the cerebellum in MDD [17], which could suggest pathophysiological involvement. Cerebellar dysfunction may contribute to abnormal expectation effects in MDD. Therefore, the cerebellum will be included in resting state network connectivity analyses. In addition, eyeblink conditioning will be performed, an associative learning paradigm that critically depends on the integrity of the cerebellum [16, 18].

In aiming to identify the different neurobiological players involved in expectations, relevant laboratory parameters should also be considered: numerous studies have demonstrated elevated inflammatory markers in unipolar depression, including CRP (C-reactive protein), IL-6 (interleukin-6), and TNFα (tumor necrosis factor α) [19]. These markers could objectify therapeutic success as an additional outcome parameter. Cortisol awakening response (CAR) is another biological marker altered in depressive disorder. In addition, CAR has been linked in brain imaging studies to networks that may also play a role in expectation effects (DMN, medial prefrontal cortex, and anterior cingulate gyrus) in such a way that it could be considered a predictor for expectation effects [20].

In summary, it is evident that patients’ expectations for a treatment represent a very important component of the placebo effect and are crucial for the response to the respective treatment [3, 21]. Expectations depend on various factors, such as personal experiences, the current situation including the context, individual attitudes, and available information [22, 23].

Not all of these factors can be influenced. Currently, it is unclear to what extent treatment expectations can be modified and how strong, and thus how relevant, such an effect could be. The study at hand aims to shed some light on this issue. Furthermore, the question arises whether the informed consent discussion could be a suitable starting point for influencing therapy expectations [24]. Should this prove to be the case, it would have promising and far-reaching implications for everyday doctor-patient communication.

TMS appears to be a particularly suitable form of treatment for investigating this matter, since placebo effects play a major role here, and moreover, the mode of action of TMS could be reinforcing precisely these results, i.e., amplifying expectancy effects.

Studies on the forms of treatment for depressive illness have in common that they show strong placebo effects, regardless of the form of treatment [25]. Expectations thus seem to play a particularly important role for the effectiveness of treatment in this disorder, not least because the impairment to develop hope and to expect something good to happen is a central symptom of this disorder. Therefore, both the disease and the form of treatment seem to be suitable for the research question of our study.

### Objectives {7}

The main objective of our study is to test the hypothesis that a higher expectation for the efficacy of TMS treatment leads to a better therapy success regarding the intensity of the depressive disorder. In this, we do not seek to investigate the efficacy of TMS in MDD per se, but rather the effects of treatment-related expectation. In order to realize this, the intervention as a more positive and comprehensive patient education aims to induce higher expectations, which, in the next step, is assumed to lead to a better outcome. Therapy success will be evaluated by means of psychometric tests and cognitive tasks. We further intend to explore a possible normalization of inflammatory parameters as an additional outcome parameter.

Furthermore, in an exploratory approach, the aim is to identify predictors of the expectancy effect in imaging, at the level of both biological and psychological parameters. Predictors of expectancy effects in functional connectivity in the rs activity in the DMN as well as in cognitive and emotional networks are to be identified. We also believe that CAR in saliva can be considered as a predictor of expectancy effects.

Another goal of our study is to investigate the role of the cerebellum in depression and expectancy effects: we hypothesize that mild cerebellar symptoms as well as a limitation in the acquisition of conditioned blink reflex responses (a cerebellum-dependent learning paradigm) can be detected in a subset of patients. Our hypothesis is that the expectancy effect is less pronounced in this group of patients, which is also reflected in the altered functional connectivity of the cerebellum.

### Trial design {8}

We are planning an interventional, monocentric, randomized, controlled trial with two MDD patient groups of 30 participants each.

Our study is of the parallel group type, i.e., we are looking at two groups of patients in parallel, with one group of patients receiving an intervention while the other one does not. The framework is “superiority”: we expect to see superiority in outcome parameters in the intervention group. The allocation ratio in the intervention versus the control group is 30:30.

As it is to be expected that about 10% of the data will be lost due to the complex study protocol with several examinations at a total of four time points, at least 35 patients should be recruited per group. This minimum recruitment number seems achievable based on our experience with previous studies. The details of the intervention are outlined below.

In addition, an age-, sex-, and education-matched control group of 30 participants will be recruited, who will serve as a control only for the additional experimental investigations, which is the MRI part and the eyeblink conditioning. In these two subprojects, the first step is to evaluate the changes in the context of depressive illness in general compared to a healthy control group, see the “Outcomes {12}” section.

## Methods: participants, interventions, and outcomes

### Study setting {9}

This is a monocentric, investigator-initiated study that will be conducted in the LVR-University-Hospital Essen, Germany, Department of Psychiatry and Psychotherapy. The LVR-University-Hospital Essen fulfills a local care mandate and is the psychiatric department of the medical faculty of the University of Duisburg-Essen. Patients are referred both regionally and trans-regionally via the Center for Treatment-Resistant Depression at LVR-University-Hospital Essen.

### Eligibility criteria {10}

Sixty patients with an MDD and 30 healthy, matched controls will be included in the study. Within the patient group, allocation to the intervention or control condition will be randomized and stratified for gender to ensure a balanced gender distribution.

The inclusion criteria are an age of 18–65 years, moderate to severe depressive episode (according to Hamilton Rating Scale for Depression, HDRS, as well as a structured clinical interview for DSM IV), and at least two different antidepressants without sufficient effect in the patient history.

The exclusion criteria include individuals under legal care and individuals in official or judicial custody, individuals with malignant diseases and/or in poor general condition, no sufficient hearing or reading ability, contraindications for TMS (that is, presence of ferromagnetic material, e.g., cochlear implants, pregnancy, cardiac pacemaker or epilepsy), previous TMS treatment, electroconvulsive therapy in the preceding 6 months, relevant structural lesions in MRI (apart from expected age-related changes such as mild cerebral microangiopathy in patients over 50 years of age or changes without expected relevance such as small arachnoid cyst), further relevant comorbidities such as substance abuse or dependence, organic mental disorders, dementia, or psychotic disorders.

In addition, the exclusion criteria relate to relevant comorbidities such as neurological disorders, e.g., multiple sclerosis, Parkinson’s syndrome, and cerebellar disease (excluding headache disorders), diseases that are associated with an inflammatory response and lead to permanently elevated inflammatory parameters and severe internal diseases that are accompanied by a significantly reduced general condition.

### Who will take informed consent? {26a}

During outpatient or inpatient treatment, potential study patients receive information about the possibility of participating in the study and are asked for permission to contact them by telephone if they are interested. In addition, interested patients who have learned about the study via the LVR-University-Hospital homepage or have received the flyer about the study via psychiatrists in a private practice can contact us via an e-mail address set up specifically for the study.

In the next step, potential participants receive further relevant information such as the time of participation and duration of the examinations via telephone. In this process, the inclusion and exclusion criteria are also systematically checked. If there are no contraindications up to this point, an appointment is made to see the investigator for an in-depth educational interview, and verbal as well as written informed consent to participate in the study is obtained.

### Additional consent provisions for collection and use of participant data and biological specimens {26b}

The participants are informed that they take part in the study voluntarily and that they can withdraw their consent to participate at any time. In addition, written consent to the collection and further processing of data in accordance with the current EU Data Protection Regulation is recorded. Since part of the data is collected in cooperation with other scientists, the participants are informed which (pseudonymized) data will be passed on to which institute and who is the responsible contact person. We will request consent for the review of participants’ medical records and for collecting blood samples to assess C395 reactive protein (CRP), interleukin (IL)-6, and tumor necrosis factor (TNF)-α, as systemic inflammatory markers will be assessed along with (epi)-genetic markers and saliva samples to target the salivary cortisol awakening response as a measure for the activity of the hypothalamic–pituitary–adrenal (HPA) axis. The participants are given the information form and a copy of the signed consent form.

Pseudonymization is realized by the ALIIAS, a software tool which implements a two-factor authenticated, de-centralized, encryption-based, deterministic pseudonymization technique to transform personal data to a pseudonym. The “full version” of the pseudonym (long ID) allows for complete re-identification (given a dedicated secret digital key owned by the “pseudonymization entity,” i.e., the individual research site). The software also provides a “human-readable” (9 characters) short ID, which is easy to link to the long ID and is compatible with most experimental procedures [26].

### Interventions

#### Explanation for the choice of comparators {6b}

In order to investigate the impact of the participants’ expectations, expectations will not be recorded retrospectively, but manipulated directly by an intervention that is described below. The treatment-as-usual condition was chosen as the control group for this study, though it must be emphasized that the term “treatment” here refers to the informed consent discussion and not the actual treatment (as explained in the “Intervention description {11a}” section).

#### Intervention description {11a}

All participants, irrespective of their assigned group, receive the same treatment with TMS within the scope of the approval. They are informed about the procedure, expected benefits, and risks. Compared to a standard briefing from a medical professional, this intervention contains more detailed and more encouraging information prior to the treatment with TMS. With this goal in mind, a separate educational sheet was designed to emphasize the promising nature of the treatment, without withholding relevant information about the risks and side effects from the participants. In this way, the intervention targets the patient’s educational process while still in the preparatory phase of an approved and established therapy. Half of the study participants (intervention group), however, receive additional information emphasizing the promising character of the therapy as well as the safety of the method. Here, we use visual material about the device, illustrations from studies that were able to prove the effectiveness of TMS and a more appealing presentation. Furthermore, an information film about TMS is presented in the intervention group only. Patients are not aware that two different types of informed consent discussions are being used.

#### Criteria for discontinuing or modifying allocated interventions {11b}

Overall, the study is at a very low risk for potential harm. No harm is expected from the intervention in the form of modified patient education about the approved therapy with TMS. Prior to the start of the study, study participants will be asked to indicate whether they would like to be informed of incidental findings in addition to this education.

The discontinuation criteria for individual participants are the occurrence of intolerable and/or unexpected undesirable side effects during the therapy with TMS, the occurrence of the exclusion criteria during the course of the therapy, violation of the study protocol, and other circumstances that would endanger the health of the participant if he or she continued to participate in the study, as well as on participant request. Change of medication during the treatment is not an exclusion criterion per se but will be recorded as an event that will need additional control.

#### Strategies to improve adherence to interventions {11c}

Participation in the study offers patients the option of treatment with TMS, which is not yet routinely available at all clinical sites in Germany and which will also be offered independently of study participation in the inpatient setting. In the outpatient setting, however, treatment costs have not always been covered by health insurance in Germany up to now, so that participation in the study may enable patients to choose a more preferable form of treatment. In addition, very extensive psychometric testing is performed, which goes beyond the standard clinical practice and can provide patients with an indication of their current level of performance. Adherence, in the present study, consists of daily attendance at the clinic where TMS is performed, which can be tracked very easily.

As therapy adherence is certainly a major challenge in the study, and in order to take quality control into account, the complexity of the protocol will already be addressed during the first telephone contact, so that the participants can adjust accordingly. In the event that at this point there is already strong uncertainty regarding the realization of the study visits, the participants will be informed about alternative treatment options for depression which would be easier to implement.

#### Relevant concomitant care permitted or prohibited during the trial {11d}

The aim is to ensure that during study participation, any existing drug and/or psychotherapeutic treatment is continued with as little change as possible. In the event of a relevant change during study participation, discontinuation must be discussed individually. Change of medication during the treatment is also not an exclusion criterion per se but will be recorded as an event that will need additional control.

#### Provisions for post-trial care {30}

The possible disadvantages (risks, impairments, burdens) for the study participants resulting from the planned examinations with MRI, blood sampling, and collection of saliva samples are negligible compared to the expected benefit. The expected risks of blood sampling are minor pain and possibly a small hematoma at the puncture site. The immediate benefit for the study participants is the possibility of an optimization of the therapy effect; in the long term, the study could mean an improvement in treatment practice. Overall, the study involves a very low risk for potential harm. No harm is expected from the intervention in the form of modified education about the approved therapy with TMS. In principle, participants will be offered the full range of depression treatment services at LVR-University-Hospital Essen as an alternative to study participation or as a future treatment option.

### Outcomes {12}

#### Primary outcomes

Psychometric tests to assess therapy success: The tests will be administered at four points in time (T1: before therapy, T2: in week 2 of therapy, T3: in week 3 of therapy, T4: after therapy). T1 was defined as the examination within a period of 2 weeks before the start of therapy, and T4 as the examination within a period of 2 weeks after the end of therapy.

The following tests are used, some of which are questionnaires to be completed by the patient and some of which record the clinical examiner’s impression.

Beck Depression Inventory-II (BDI-II): This instrument measures the severity of depressive symptoms. In the form of a questionnaire, the patient self-reports how pronounced certain symptoms were during the previous week [27].

Montgomery Asberg Depression Scale [MADRS [28]]: In contrast to the BDI-II, MADRS is an instrument for third-party assessment of the severity of a depressive episode. The questionnaire is completed by the patient’s clinical practitioner as part of the usual clinical routine or by a clinical psychologist, who is blinded to intervention allocation.

Clinical Global Impression severity scale (CGI): This is a brief third-party assessment of the severity of illness by the examiner [29, 30], who is blinded to intervention allocation.

Snaith-Hamilton Pleasure Scale: This questionnaire is used to measure subjectively experienced joylessness (anhedonia) in psychiatric patients [31, 32].

#### Secondary outcomes

Psychometric tests to assess therapy expectation: The tests will be administered at four points in time (T1: before therapy, T2: in week 2 of therapy, T3: in week 3 of therapy, T4: after therapy).

Treatment Expectation Questionnaire (TEX): In this questionnaire, study participants are asked to self-assess their expectations for a treatment in 15 questions on a scale of 1–10 [33].

Generic rating for treatment pre-Experiences, treatment Expectations, and treatment Effects (G-EEE): The questionnaire is used as a self-report instrument to assess the expectations for a treatment, pre-experiences, and treatment effects. The questionnaire will be applied to treatment with TMS in the present study.

Structured Clinical Interview for DSM-V (SCID): This is a common procedure for diagnosing mental disorders according to the DSM-V classification system of psychiatry [34]. It is to ensure that no relevant comorbidities are present.

Generic Assessment of Side Effects (GASE): This questionnaire assesses side effects [35] and will only be applied at T4 with the aim to record side effects that occurred during the course of the treatment.

Wechsler Adult Intelligence Scale (WAIS) IV Vocabulary Test: The vocabulary subtest of the WAIS IV [36] is designed to roughly estimate participants’ crystalline intelligence, i.e., acquired cognitive skills. The “vocabulary test” is the second core test of the index language comprehension. Individuals name objects presented as pictures or explain concepts presented orally or in writing. It measures a person’s vocabulary and concept formation.

Number Connection Test: This test [37] is a language-free intelligence test designed to roughly assess the cognitive flexibility of test participants.

Experimental computer-based test procedures for recording the success of the therapy: survey at two points in time, before and after the therapy.

Movie for the Assessment of Social Cognition (MASC): The MASC [38] is a well-validated instrument for assessing social cognition, in particular the ability to grasp the thoughts, feelings, and intentions of other people. As this ability is diminished in people with (chronic) depression [39–41] and the limitations in social cognition may themselves contribute to maintaining depressive symptomatology [42], social cognition is also an important measure of the success of sustained antidepressant treatment. To measure this, participants are shown a 15-min video of four friends meeting for dinner. The video is stopped a total of 46 times, so that the participants can answer questions about the situation presented. The participants have to consider different sources of information (language, facial expressions, gestures), and the sequences shown have different valences (negative/neutral/positive) and are of varying complexity (some questions require an understanding of sarcasm or metaphors, for example).

Trust game: In accordance with the reduced ability of depressed individuals to identify thoughts, feelings, and intentions in others, as described above, depression is thought to involve reduced emotional mimicry (i.e., imitating social cues of the other person with the goal of building trust) [43]. Based on the described deficits in emotion recognition, depressed patients also have more difficulties trusting other people [43]. Participants have to invest money in a virtual counterpart which either reflects the emotional expression of the participant or not. The outcome parameter is the investment behavior of the participants.

Emotion recognition task: This task aims at the reduced ability of depressive patients to recognize emotions in other people [40]. For this task, participants will be shown a selection of 140 photos of ten models (five women, five men). These are taken from the Karolinska Directed Emotional Faces [KDEF [44]], with each model showing seven facial expressions (happy, surprised, angry, sad, fearful, disgusted, and neutral) both frontally and in profile. In the task, participants are shown a facial expression for a duration of 700 ms, after which they must name the expression as accurately and quickly as possible, with responses and reaction times recorded as outcome parameters.

Biological parameters (neuroimmunology, (epi-)genetics): Survey at two time points (T1: before therapy, T4: after therapy).

Blood sampling: In total, less than 60 ml of blood will be taken. Plasma concentrations of C-reactive protein (CRP), interleukin (IL)-6, and tumor necrosis factor (TNF)-α as systemic inflammatory markers will be determined along with (epi)-genetic markers, e.g., acetylations.

Collection of saliva: Saliva samples will be self-collected by the study participants with commercial collection devices. This is a painless procedure that usually takes 60 s. The salivary cortisol awakening response will be used as a measure of the activity of the hypothalamic–pituitary–adrenal (HPA) axis. Hormone determinations will require three saliva samples taken on each of two consecutive days immediately, 30 and 45 min after awakening.

Parameters indicative of cerebellar dysfunction: survey at one time point before therapy (neurological examination and ataxia scores, see below).

Eyeblink conditioning: Delay conditioning paradigm will be used [16]. A tone will be used as the conditioned stimulus (CS) and an air puff directed to the outer canthus of the eye as the unconditioned stimulus (US). CS and US coterminate. Surface EMG recording of the orbicularis oculi muscles will be used to measure unconditioned (UR) and conditioned (CR) responses. The focus will be on the acquisition of conditioned blink reflex responses [16, 18]. Participants learn that the CS predicts the occurrence of the US and learn to close their eyes prior to the occurrence of the US. The CR incidence is the primary outcome parameter and will be determined using semi-automated analysis software. CR timing will also be assessed.

Imaging: Functional connectivity in resting state MRI and diffusion tensor imaging (DTI) before and after therapy.

MRI of the skull: The examination takes place in a 3 T scanner (Magnetom Vida, Siemens, Erlangen, Germany, 3 T) at the University Medical Center Essen (Institute for Diagnostic and Interventional Radiology).

Functional imaging–resting-state: fMRI allows indirect measurement of neuronal-related changes in brain metabolic activity (blood oxygenation level-dependent (BOLD) response). The advantages of the method include the recording of the activity of the entire brain in a few seconds, as well as the spatial resolution achieved in the process. During the rs measurement, the participants are not confronted with any tasks or stimulation beyond “lying quietly.”

Structural recordings: High-resolution, structural, T1-weighted recordings of the brain are required for the analysis of the fMRI data and are acquired using MP-RAGE sampling (or similar T1-weighted methods). Participants have no other tasks to perform during the examinations.

Diffusion tensor imaging (DTI): DTI sequences are applied to investigate structural connectivity in addition to predictors of expectancy effects at the level of functional connectivity. The measurement time for the complete protocol is approximately 30 min.

For the last four items (i.e., experimental computer-based test procedures, biological parameters, eyeblink conditioning, and MRI), the healthy control group is needed as explained in section “Trial design {8}”.

### Additional measures

Edinburgh Handedness Inventory: This is a short questionnaire designed to assess handedness [45].

### Participant timeline {13}


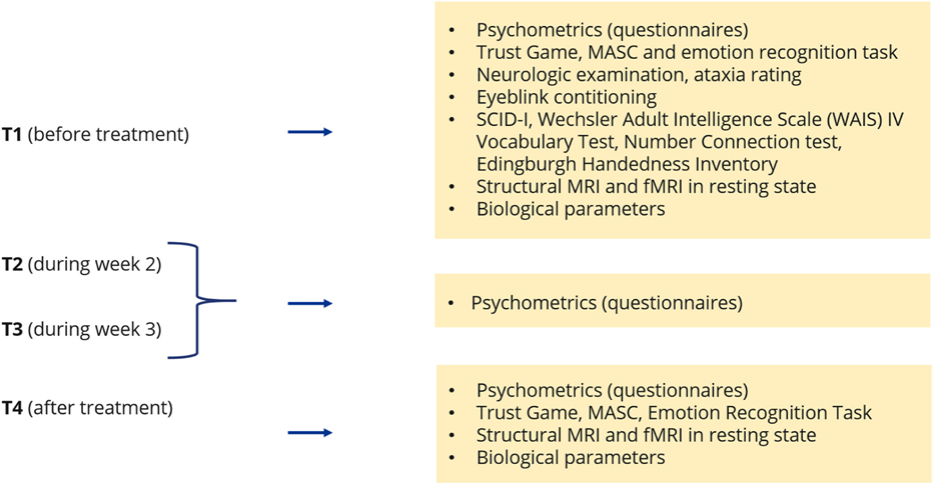


### Sample size {14}

Since no study with the same or similar content has been conducted to date, the case number calculation was based on a study by Claus et al. [7], who also investigated the expectancy effect with regard to antidepressant pharmacotherapy. In this study, the outcome was measured by the BDI and HRSD, CGI, SHPS, and Well-Being Questionnaire WHO-5 that showed effect strengths ranging from 0.79 to 1.6 (Cohen’s *d*; repeated-measures ANOVA, four time points, *α* = 0.05, 1 − *β* = 0.95, two groups). A case number calculation based on these effect sizes yielded a case number of *n* = 7 per intervention group. On the other hand, it must be emphasized at this point that in studies on antidepressant treatment, mostly small effect sizes are observed, which we do not want to disregard. To account for an effect size in the current study that is expected to be lower than the one in the work by Claus et al., the necessary sample size was estimated to be an *n* = 30.

As it is to be expected that due to the complex study protocol with several examinations at a total of four time points, about 10% of the data will be lost, at least 35 patients per group should be recruited.

### Recruitment {15}

Through the newly established Center for Treatment-Resistant Depression (*Zentrum für Therapieresistente Depressionen*) at the LVR-University-Hospital Essen, a large number of interested and/or referred patients present themselves for a consultation. Furthermore, a flyer was created that summarizes the content of the study to be distributed to psychiatric practices. In addition, a training event on the topic of therapy-resistant depression will be conducted to familiarize potential referring physicians with the study. The recruitment of the healthy control group is based on age, gender, and education of the patient group. For this purpose, the study team recruits control subjects from the professional and private environments. In addition, an appeal via social media is planned in case not enough participants are found via the first step. The recruitment period is scheduled for a period of 1 year.

### Assignment of interventions: allocation

#### Sequence generation {16a}

The participants are divided into two groups (intervention group versus control group) with an allocation ratio of 1:1 and *n* = 30 participants per group. Stratification by gender is performed to ensure a balanced gender ratio; beyond that, there are no other factors used for stratification. The freely available online software “randomizer” (randomizer.org; last access 8/15/2023) will be used for this purpose.

#### Concealment mechanism {16b}

The allocation order is implemented and concealed by the randomization tool “randomizer” (randomizer.org; last access 8/15/2023). The allocation sequence is stored in a password-protected folder.

#### Implementation {16c}

Sealed envelopes are used to store the randomization result for the individual participant. These are opened only immediately before the intervention by the investigator.

### Assignment of interventions: blinding

#### Who will be blinded {17a}

This is a double-blinded study, i.e., the participants do not know which group they were assigned to, nor that an intervention takes place during the educational process. Since the investigator performs the medical consultation, which at the same time is the intervention, blinding is not possible here. The additional scientific staff who survey the outcomes (i.e., eyeblink conditioning and cognitive tasks) are blinded regarding the intervention group during the entire course of the study. As the therapist factor should not be underestimated, the protocol is realized in a way that all the patients interact with the same therapist for each respective procedure (the same person performing the psychological tests and interviews, the same person applying the TMS treatment, and the same person communicating regarding the informed consent).

#### Procedure for unblinding if needed {17b}

Since the intervention is not an alternative treatment, there are no conceivable reasons that would necessitate unblinding.

### Data collection and management

#### Plans for assessment and collection of outcomes {18a}

A description of the study instruments including corresponding references can be found in the “Outcomes {12}” section. All the staff involved in the data collection will be trained accordingly prior to the start of the study; furthermore, the first investigations performed by staff will take place under the supervision of the principal investigators.

#### Plans to promote participant retention and complete follow-up {18b}

The participants are informed that another detailed test will take place at the time point T4 after the treatment; if desired, they will be informed about the point values of the questionnaires. Furthermore, there is a genuine motivation of the participants to receive the treatment at a sufficient length. In case of discontinuation or deviation from the study protocol, the reason(s) for discontinuation will be recorded and the tests that would have been scheduled at T4 will take place at this time.

#### Data management {19}

The experimental data (questionnaires, computer-based test procedures, MRI data, sample IDs) are given a letter/number code, i.e., they are pseudonymized (procedure described above). The data are stored on a local PC. The evaluation is also carried out on the local PC.

The blood and DNA samples will be stored (under the pseudonymized code) in a monitored and lockable − 20 °C or − 80 °C freezer in secured laboratory rooms accessible only to authorized laboratory staff. The blood and saliva samples are immediately centrifuged and frozen at − 20 °C or − 80 °C until further analysis. The determination of inflammatory markers and CAR is performed by the Institute of Medical Psychology at the University of Duisburg-Essen. The determination of CRP is done by immunoassays, and the determination of IL-6 and TNFα as well as CAR by ELISA. Storage of the samples for future use is not planned.

The structural and functional cranial MRI data acquired during the MRI measurement are additionally stored in the electronic archive of the Institute of Interventional Radiology and Neuroradiology at the University Hospital Essen, where they are evaluated and archived by the neuroradiologists involved in the study.

Within the framework of the cooperation, collected data (e.g., clinical scores, eyeblink conditioning data, DTI data, (epi-)genetic and neuroimmunological data) as well as general personal data (e.g., gender, age, education) are exchanged between the research groups in a pseudonymized form.

#### Confidentiality {27}

All examination results are treated strictly confidentially. Person-identifying data (name, date of birth, contact details, written informed consent) are recorded in paper form. Furthermore, pre-existing conditions, previous treatments, and medications taken are recorded. Patients are asked for copies of out-of-pocket medical records if necessary. All person-identifying paper records are kept in a locked cabinet at the LVR-University-Hospital Essen. Only authorized members of the working group have access to the laboratory and the cabinets. The experimental data (questionnaires, computer-based test procedures, MRI data, sample IDs) are given a letter/number code, i.e., they are pseudonymized (procedure described above). The data are stored on a local PC, which is protected by a password. The pseudonymization list, via which an assignment of the number code to the person-identifying data is possible, is stored separately from the other documents in a lockable cabinet. All personal identifying data collected in the experiment, including the signed consent forms, will be kept until at least the end of the study and for a maximum of 10 years. Thereafter, all personal-identifying data, including the encryption list and the written informed consent form, will be destroyed. The remaining data is then stored permanently in anonymized form. Only the principal investigators and members of the working group have access to the local PC and the lockable cabinet. Pseudonymization means, as mentioned above, that name and date of birth are replaced by a code and their assignment to the individual participant is restricted to the staff members of this study. The participants are informed that the collected data will be published in pseudonymized (and in the course anonymized) form in scientific journals. They are also informed that pseudonymized or anonymized data can be made available to other scientists, e.g., by publishing such data on scientific data platforms or at the request of other scientists. The participants are informed about their rights according to the European Data Protection Regulation (EU-DSGVO), i.e., the right to information about the processing of their data, correction or deletion of their data, restriction of processing (only storage possible), objection to processing, data portability, revocation of their given consent with effect for the future, and complaint to the data protection supervisory authority. Participants are informed that they have a right to have their data deleted at request. They are also informed that data, once anonymized, can no longer be destroyed.

#### Plans for collection, laboratory evaluation, and storage of biological specimens for genetic or molecular analysis in this trial/future use {33}

The collection, evaluation, and storage of biological samples are carried out exclusively within the framework of cooperation with the Institute of Medical Psychology and Behavioral Immunobiology (Prof. H. Engler) at the University of Duisburg-Essen and the Department for Psychiatry, University Hospital Frankfurt (Prof. J. Repple). The blood and saliva samples are immediately centrifuged and frozen at − 20 °C until further analysis. The determination of inflammatory markers and CAR is performed by the Institute of Medical Psychology at the University of Duisburg-Essen. The determination of CRP is done by immunoassays and the determination of IL-6 and TNFα as well as CAR by ELISA. Storage of the samples for future use is not planned.

## Statistical methods

### Statistical methods for primary and secondary outcomes {20a}

Analysis of variance (ANOVA) with repeated measures is planned for the evaluation of the parameters of therapy outcome (questionnaires for depression), therapy expectancy (questionnaires see above), biological parameters, and tests for cerebellar function (neurological examination, ataxia scores, and blink reflex conditioning). In case of missing values, a linear mixed model analysis is planned. An unpaired *T*-test or chi-square test is planned to determine the group differences at a given point in time. Correlation analyses are planned to correlate parameters of therapy success with treatment expectations.

The MRI data will be analyzed using statistical parametric mapping (SPM, https://www.fil.ion.ucl.ac.uk/spm/; last access 8/15/2023): automated group comparisons in terms of *T*-test and ANOVA will be performed including covariates.

### Interim analyses {21b}

Interim analysis is not provided.

### Methods for additional analyses (e.g., subgroup analyses) {20b}

No subgroup analyses are planned.

### Methods in analysis to handle protocol non-adherence and any statistical methods to handle missing data {20c}

Linear mixed model analyses are planned in case of missing values.

### Plans to give access to the full protocol, participant-level data, and statistical code {31c}

There are no plans for granting public access during the run-up to publication. The datasets analyzed during the current study and statistical code are available from the corresponding author on reasonable request, as is the full protocol.

### Oversight and monitoring

#### Composition of the coordinating center and trial steering committee {5d}

This is a monocentric, investigator-initiated study. The coordinating center is the LVR-University-Hospital Essen. The daily local organization and coordination of the study are ensured by Dr. Katharina Marie Steiner (KMS), who obtains informed consent as the clinic’s physician. Prof. Dr. Katja Koelkebeck (KK) and Prof. Dr. Dagmar Timmann-Braun (DT) supervise the study. Meetings are held regularly every 3 months as part of the in-house research conference and at short notice to clarify questions and problems that may arise. There is no Stakeholder and Public Involvement Group (SPIG).

#### Composition of the data monitoring committee, its role, and reporting structure {21a}

This is a monocentric, investigator-initiated study. No data monitoring committee was required by the local Ethics Committee as this is a low-risk intervention.

#### Adverse event reporting and harms {22}

An adverse event (AE) can be an unfavorable finding, a new symptom, or a new disease that is temporally related to the study participation. These are systematically recorded with the date of occurrence; in the event of further study participation, the course is documented accordingly. Conceivable adverse events and serious adverse events in the course of the study are, first and foremost, a worsening of the depressive disorder, as can occur in the natural course of the disease. Complications such as acute suicidality are conceivable, which could necessitate emergency hospitalization. A connection with the study cannot be assumed, since the intervention is not a treatment in the strict sense. Detailed documentation of all conceivable AEs and/or SAEs is provided. The assessment of causality and expectedness is carried out by KMS and KK. A causal relationship is to be excluded due to the character of the intervention.

#### Frequency and plans for auditing trial conduct {23}

As this is a very low-risk intervention, there are no explicit audits, but there is a regular exchange based on the initiative of the Trial Steering Group with the local ethics committee regarding the status of the study. Meetings of the Project Management Group and the Trial Steering Group are held regularly every 3 months as part of the in-house research conference and at short notice to clarify questions and problems that may arise.

#### Plans for communicating important protocol amendments to relevant parties (e.g., trial participants, ethical committees) {25}

In case of changes to the study protocol, these are submitted to the local ethics committee in the form of an official amendment and reported to the German Registry of Clinical Trials (DRKS; https://www.drks.de/drks_web/; last access 8/15/2023).

#### Dissemination plans {31a}

Publication of the results of the study in a peer-reviewed journal as well as the presentation of posters or talks on the (preliminary) outcomes are planned. There are no publication restrictions.

## Discussion

The aim of the present study is to investigate the influence of the expectations of patients with therapy-resistant depression on the treatment with TMS. The therapy success will be objectified by cognitive instruments and biological parameters in addition to instruments of self- and external assessment of depressive symptoms. Furthermore, the neuronal networks relevant to therapy expectancy will be investigated in order to understand underlying mechanisms more closely and to be able to optimize the possibilities of a well-tolerated non-invasive treatment in the long term. In particular, the role of the cerebellum will be investigated, as it might contribute to treatment expectations. For the evaluation of cerebellar function, eyeblink conditioning will be used in addition to a clinical examination. In eyeblink conditioning, the ability to predict future events is central for learning to occur and depends on the integrity of the cerebellum.

Using analysis of functional connectivity with rs data, predictors of the expectancy effect will be identified. It is known from the literature that these overlap with predictors of treatment response as well as stimulation targets of treatment of depression by TMS or DBS [12].

Thus, the present study aims at contributing to the question to what extent the effect of treatment of MDD by noninvasive stimulation can be separated from an influence on treatment expectation in general, in terms of the placebo effect. This could be the starting point for a deeper understanding of the pathophysiology of depressive disorder. Conversely, the principle of enhancing expectancy effects by noninvasive stimulation could be transferable to other disease patterns.

A weakness of this study may be the low case number. For our power calculation, studies investigating treatment expectancy in other disorders [46] were considered in addition to the above-mentioned study by Claus et al. [7], who investigated a psychological intervention in addition to antidepressant medication. In particular, the generally low effect sizes of antidepressant treatments [47] could be problematic here.

Another difficulty is certainly the complex and in part very extensive study protocol, which not only might limit the number of study participants but can also amount to a considerable effort for patients suffering from depressive disorder. In order to accommodate the study participants, it is possible to distribute the examinations planned before the start of the study over several appointments. Nevertheless, this aspect, as well as the high frequency of treatment days, could discourage participants with long travel distances from participating in the study.

Despite this, the study offers the possibility to increase the availability of treatment with TMS, a comparatively new and not yet widely introduced treatment for therapy-resistant depression, also on an outpatient basis. Furthermore, the study should contribute to the understanding and treatment optimization of an individually very disabling and economically highly relevant disease.

## Trial status

This is the first version of the study protocol (2022/07/01). Recruitment started in June 2022 with the goal of completing recruitment by November 2023.

## Data Availability

After publication in a peer-reviewed journal, the dataset will be made available to other scientists upon personal request and justified intention.

## Abbreviations

AE: Adverse events
BDI: Beck depression inventory
CAR: Cortisol awakening response
CRP: C-reactive protein
DBS: Deep brain stimulation
DMN: Default mode network
DRKS: German Registry of Clinical Studies
DTI: Diffusion tensor imaging
EU-DSGVO: European Data Protection Regulation
(f)MRI: (Functional) magnetic resonance imaging
HDRS: Hamilton Rating Scale for Depression
IL-6: Interleukin 6
MDD: Major depressive disorder
(r)TMS: (Repetitive) transcranial magnetic stimulation
RS: Resting state
TNFα: Tumor necrosis factor α
